# Probing the pathogenicity of patient-derived variants of *MT-ATP6* in yeast

**DOI:** 10.1242/dmm.049783

**Published:** 2023-04-21

**Authors:** Emilia Baranowska, Katarzyna Niedzwiecka, Chiranjit Panja, Camille Charles, Alain Dautant, Jarosław Poznanski, Jean-Paul di Rago, Déborah Tribouillard-Tanvier, Roza Kucharczyk

**Affiliations:** ^1^ Institute of Biochemistry and Biophysics, Polish Academy of Sciences, 02106 Warsaw, Poland; ^2^ University of Bordeaux, Centre National de la Recherche Scientifique, Institut de Biochimie et Génétique Cellulaires, UMR 5095, F-33000 Bordeaux, France

**Keywords:** Mitochondrial diseases, ATP synthase, Yeast, Mitochondrial DNA mutation, MILS, LHON, mtDNA, *MT-ATP6*

## Abstract

The list of mitochondrial DNA (mtDNA) variants detected in individuals with neurodegenerative diseases is constantly growing. Evaluating their functional consequences and pathogenicity is not easy, especially when they are found in only a limited number of patients together with wild-type mtDNA (heteroplasmy). Owing to its amenability to mitochondrial genetic transformation and incapacity to stably maintain heteroplasmy, and the strong evolutionary conservation of the proteins encoded in mitochondria, *Saccharomyces cerevisiae* provides a convenient model to investigate the functional consequences of human mtDNA variants. We herein report the construction and energy-transducing properties of yeast models of eight *MT-ATP6* gene variants identified in patients with various disorders: m.8843T>C, m.8950G>A, m.9016A>G, m.9025G>A, m.9029A>G, m.9058A>G, m.9139G>A and m.9160T>C. Significant defect in growth dependent on respiration and deficits in ATP production were observed in yeast models of m.8950G>A, m.9025G>A and m.9029A>G, providing evidence of pathogenicity for these variants. Yeast models of the five other variants showed very mild, if any, effect on mitochondrial function, suggesting that the variants do not have, at least alone, the potential to compromise human health.

## Teaser

**Summary:** Yeast studies provide biochemical data arguing for the pathogenicity of m.8950G>A, m.9025G>A and m.9029A>G, and neutral character of m.8843T>C, m.9016A>G, m.9058A>G, m.9139G>A and m.9160T>C, variants in *MT-ATP6*.

## INTRODUCTION

Mitochondrial diseases are a broad group of neuromuscular and metabolic disorders resulting from defects in oxidative phosphorylation, a process that provides cells with the energy-rich ATP molecule (Gorman et al., 2016). This process typically involves five protein complexes (I-V) embedded in the mitochondrial inner membrane that transfer electrons from food to oxygen coupled to production of ATP from ADP and inorganic phosphate (Saraste, 1999). Of the 90 subunits present in these complexes, 13 subunits are encoded by the mitochondrial genome [mitochondrial DNA (mtDNA)], while the others have a nuclear genetic origin; therefore, mutations in both genomes can cause these diseases.

Mutations of the mitochondrial genome are more frequent that those of the nuclear genome, possibly because of its exposure to mutagenic reactive oxygen species produced inside the organelle. Variations of this genome lead to mitochondrial diseases, and are also associated with common complex diseases like Alzheimer's disease, Parkinson's disease, Huntington diseases, diabetes, cancer, and a spectrum of nonspecific features like fatigue, deafness, multiple organ failure, schizophrenia and autism (El-Hattab et al., 2014; Hudson et al., 2014; Ju et al., 2014; Pfeffer and Chinnery, 2013; Wallace, 2010). With the advent of novel genomic sequencing methods, the list of such mutations is continuously expanding (Al-Kafaji et al., 2022; Wang et al., 2022). Evaluating their functional consequences and pathogenicity is not an easy task, especially when they are found in only a limited number of patients, sometimes a single individual, and when they coexist in cells and tissues with the wild-type mtDNA, which is referred to as heteroplasmy. The homoplasmic cybrid cell lines derived from patient cells are a reliable model for the evaluation of pathogenic effect of mtDNA variants. However, the nuclear background of the ρ^0^ cell line generated to create the cybrid lines, often being the tumor cell line, contributes to conflicting findings (Wilkins et al., 2014). Recently, new mtDNA editing methods in mammalian cells have been developed; however, they are limited to specific nucleotide changes (Cho et al., 2022; Lei et al., 2022; Mok et al., 2020).

These difficulties led investigators to use the yeast *Saccharomyces cerevisiae* as a model to evaluate the consequences of mtDNA mutations found in patients. Owing to its good fermenting capacity, this unicellular fungus can efficiently survive mutations that inactivate oxidative phosphorylation, its mitochondrial genome can be modified [using a biolistic particle delivery system (Bonnefoy and Fox, 2001)], and heteroplasmy is highly unstable in this organism, making it possible to isolate strains homoplasmic for a given mtDNA mutation. Taking advantage of these attributes, we created yeast models of mutations in the *MT-ATP6* gene, encoding ATP synthase membrane subunit 6, with a proven or suspected pathogenicity (Kabala et al., 2014; Kucharczyk et al., 2019b, 2010, 2013, 2009a,b; Skoczen et al., 2018). All led to significant ATP production deficits in yeast mitochondria due to impairment of the functioning or assembly of the ATP synthase complex (Complex V) in which the protein encoded by *MT-ATP6* (subunit *a*) is essential for moving protons across the mitochondrial inner membrane coupled to ATP synthesis. Those mutations responsible for severe clinical phenotypes proved to affect dramatically the yeast ATP synthase, whereas those leading to milder diseases compromised oxidative phosphorylation in yeast mitochondria much less severely (Kucharczyk et al., 2009a,b), which validates the use of yeast as a model system to investigate the consequences of specific mutations in the human subunit *a*.

We here report the functional investigation, in yeast, of eight novel *MT-ATP6* variants detected recently in patients with various disorders: m.8843T>C, m.8950G>A, m.9016A>G, m.9025G>A, m.9029A>G, m.9058A>G, m.9139G>A and m.9160T>C. The results indicate that three of them (m.8950G>A, m.9025G>A and m.9029A>G) significantly compromise ATP synthase function, whereas the five others have very minor, if any, effect, suggesting that they do not have the potential, at least alone, to compromise human health.

## RESULTS

### Creation of yeast strains with equivalents of the human *MT-ATP6* gene mutations

**Fig. 1.**
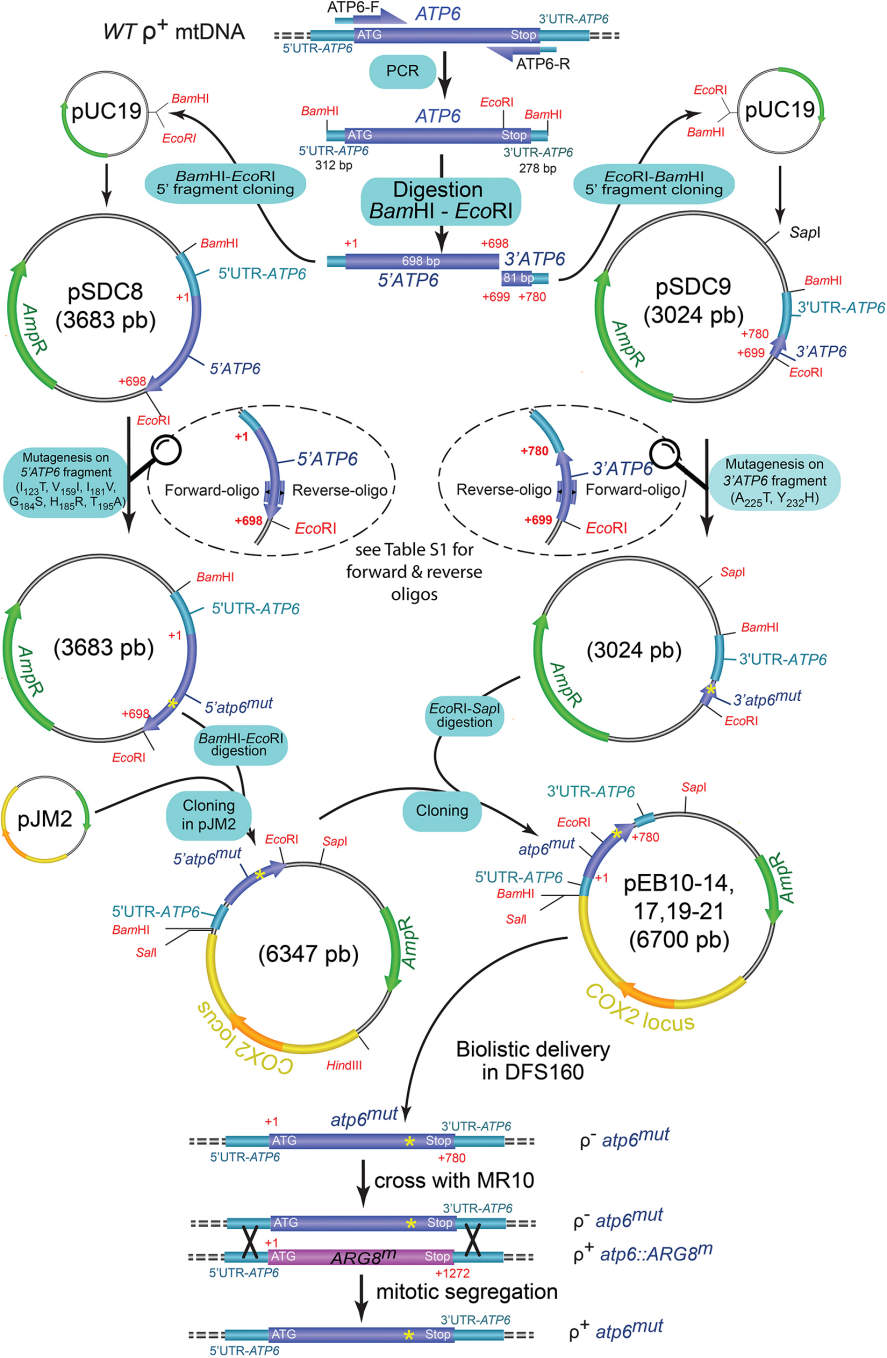
**Schema of the procedure used to create the ρ^+^ *atp6^mut^* strains.** The mutations (*atp6^mu^*^t^) are first introduced in one of two plasmids carrying a 5′ (pSDC8) or 3′ (pSDC9) fragment of the yeast *ATP6* gene. The 5′ fragment (mutated or non-mutated) is cleaved and cloned in pJM2 and is then joined with the 3′ fragment (mutated or non-mutated) using the indicated restriction enzymes to reconstitute the entire *ATP6* gene with the *atp6^mut^* mutation. The resulting plasmids (pEB) are delivered with a biolistic system in the DNA-less (ρ^0^) mitochondria of strain DFS160, giving the synthetic strains ρ^−^ atp6^mut^. These are crossed with a strain (MR10) in which the coding sequence of *ATP6* is replaced by *ARG8^m^*. In the mated cells, *ARG8^m^* is replaced by the mutated *atp6* gene by DNA recombination, and, after a dozen mitotic divisions, cells homoplasmic for the *atp6^mu^*^t^ mutation in a complete (ρ^+^) mtDNA emerge.

The eight variants of the human *MT-ATP6* gene investigated in this study led to replacement of conserved residues of subunit *a*: p.I_106_T (m.8843T>C), p.V_142_I (m.8950G>A), p.I_164_V (m.9016A>G), p.G_167_S (m.9025G>A), p.H_168_R (m.9029A>G), p.T_178_A (m.9058A>G), p.A_205_T (m.9139G>A) and p.Y_212_H (m.9160T>C) (Table 1). The corresponding changes in the yeast subunit *a* (or Atp6) are, respectively, *a*I_123_T, *a*V_159_I, *a*I_181_V, *a*G_184_S, *a*H_185_R, *a*T_195_A, *a*A_225_T and *a*Y_232_H (see Fig. 5A for amino acid sequence alignments). These mutations were first introduced into the yeast *ATP6* gene carried by a plasmid (see Table 1 for the corresponding codon changes) and then delivered into the DNA-less (ρ^0^) mitochondria of strain DFS160 with a biolistic system, giving the synthetic ρ^−^ *atp6*^mut^ strains (Fig. 1). These were crossed on glucose plates with layers of cells from strain MR10 (Rak et al., 2007b), in which the coding sequence of *ATP6* is replaced by *ARG8^m^* [this is a mitochondrial version of a nuclear gene (*ARG8*) that encodes a mitochondrial protein (Arg8) involved in arginine biosynthesis (Steele et al., 1996)]. In ρ^−^ *atp6*^mut^×MR10 (*atp6::ARG8^m^*) zygotic cells, the variant of the *ATP6* gene can be integrated into a complete (ρ^+^) mitochondrial genome by mtDNA recombination. The modified mitochondrial genome then segregates to homoplasmy in about a dozen mitotic divisions (without any selection pressure in rich 10% glucose), giving the strains ρ^+^ *atp6^mut^* used in the experiments described below. If the *atp6* mutation does not fully inactivate the ATP synthase, the mutant cells can be isolated by virtue of their ability to grow (even slowly) on respiratory carbon sources like glycerol. The eight ρ^−^ *atp6*^mut^×MR10 crosses produced respiring clones, and, as expected, DNA sequencing confirmed that these carried the *atp6* mutations. Owing to the presence in DFS160 of the nuclear karyogamy delaying *kar1-1* mutation, the *atp6* mutations could be isolated in the haploid nuclear genetic background of MR10 (MR10 is derived from wild-type strain MR6) (Table S2).

**Table 1.**
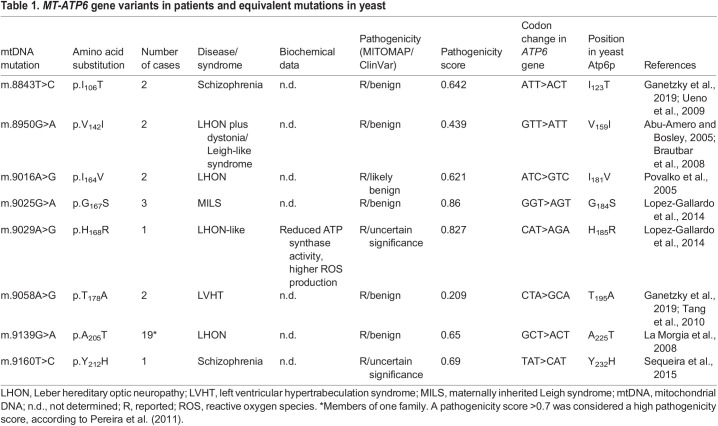
*MT-ATP6* gene variants in patients and equivalent mutations in yeast

### Influence of the *atp6* mutations on the respiration-dependent growth of yeast

#### On solid media

**Fig. 2.**
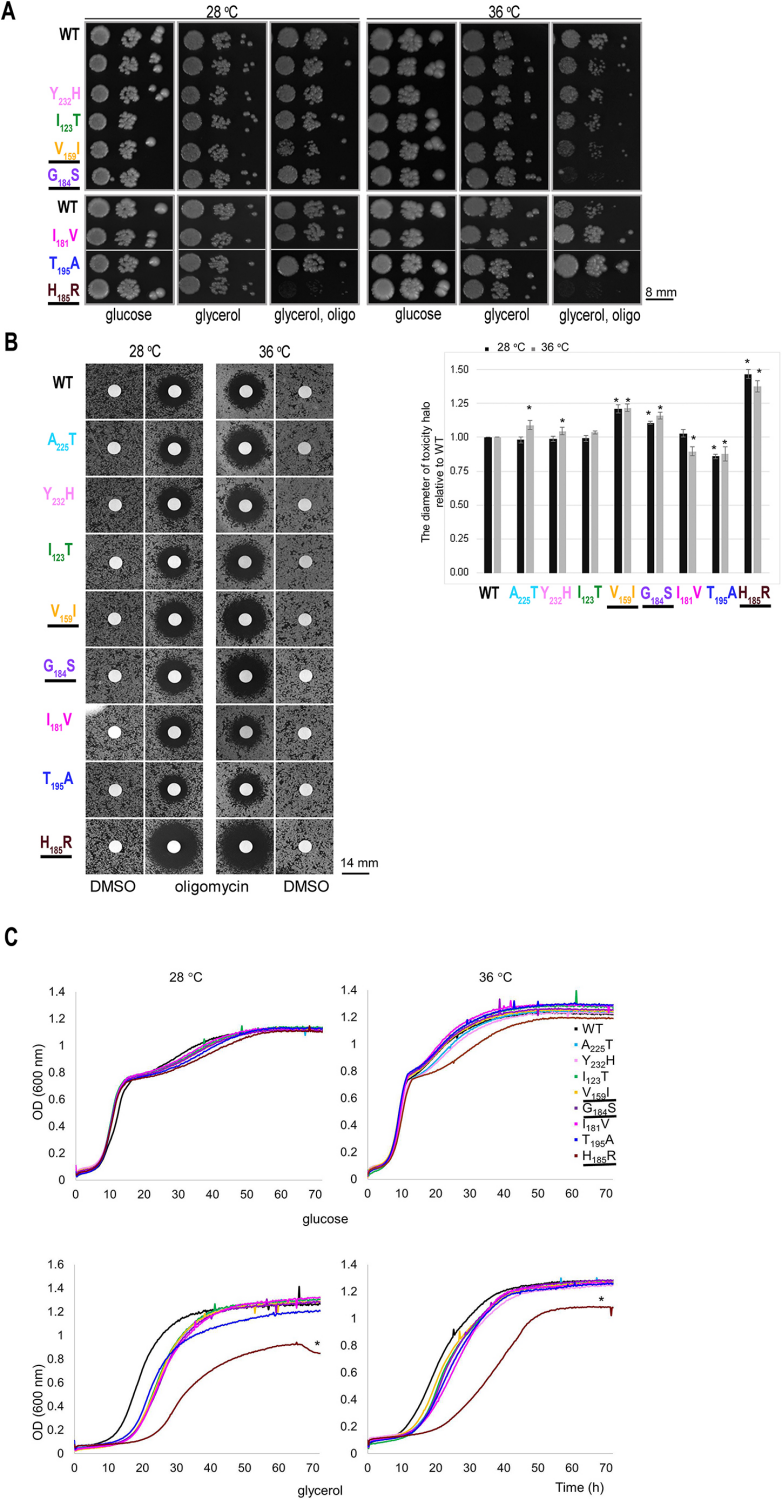
**Influence of *atp6* mutations on the respiration-dependent growth of yeast.** (A) Fresh glucose cultures of the *atp6* mutants and wild-type yeast were serially diluted and spotted on rich glucose and glycerol plates supplemented or not with oligomycin (0.5 μg/ml) and incubated at 28°C or 36°C. The glucose and glycerol plates were photographed after 3 days of incubation, while the glycerol plates supplemented with oligomycin were incubated for one more day before being photographed. Gray borders demarcate strains grown on the same plate. Strains for which growth is different from that of the control strain are underlined. WT, wild type. (B) Cells from the indicated strains were spread as dense layers onto rich glycerol solid media and then exposed to sterile filters spotted with 20 nmol oligomycin and DMSO as a negative control (solvent). The plates were scanned after 4 days of incubation at 28°C and 36°C. The diameters of the halos of growth inhibition (in % of WT) are reported in the shown histograms. *P<0.001 (unpaired two-tailed Student's *t*-test). (C) Growth in liquid glucose and glycerol media. The cultures were inoculated with cells freshly grown in glucose and monitored with a Bioscreen CTM system. *P<0.05 (unpaired two-tailed Student's *t*-test). The shown data are representative of three independent experiments. OD, optical density.

As expected, the eight yeast *atp6* mutants grew well with fermentation of glucose, conditions under which ATP synthase function is not required (Fig. 2A). They grew quite well on respiratory carbon sources like glycerol, both at 28°C (the optimal temperature for yeast) and 36°C [at this temperature yeast mitochondrial function is partially compromised (Yamamura et al., 1988)]. This did not mean that the *atp6* mutations had no deleterious consequences. Indeed, large decreases in ATP synthase activity (∼80%) are required to significantly affect the growth of yeast cells on non-fermentable substrates (Mukhopadhyay et al., 1994). However, with <80% ATP production deficits, respiration-dependent growth of yeast becomes more sensitive to oligomycin, a specific inhibitor of ATP synthase, because less of this drug is then needed to reach the ATP synthase activity threshold (Kucharczyk et al., 2010). As shown in Fig. 2A, at a concentration of oligomycin (0.5 μg/ml) that had no effect on wild-type yeast, the respiration-dependent growth of two mutants (*a*H_185_R and *a*G_184_S) was fully inhibited at 28°C and 36°C (*a*H_185_R), or mainly at 36°C (*a*G_184_S), growth of *a*V_159_I was slightly inhibited, and growth of the five other mutants was preserved. The sensitivity of growth to oligomycin was further quantified. Cells were spread as a dense layer on glycerol medium and then exposed to a drop of oligomycin deposited on a sterile filter (Fig. 2B). Oligomycin diffuses in the growth medium, which results in the establishment of a continuous gradient around the filters. Growth is inhibited until a certain drug concentration, which manifests as a halo of no growth around the filter, the diameter of which can be measured. The halos of growth inhibition had a much higher diameter for *a*H_185_R, *a*G_184_S and *a*V_159_I versus that of wild-type yeast (Fig. 2B), further indicating that these three substitutions have detrimental effects on ATP synthase.

#### In liquid media

To better appreciate the influence of the *atp6* mutations on the growth of yeast, we followed changes in cellular density over time in rich glucose and glycerol liquid media, at 28°C and 36°C. In liquid glucose, the cells first multiplied rapidly by fermentation and then more slowly by respiring the ethanol that resulted from glucose fermentation in the rapid growth phase. Although there was no obvious difference between the mutants and the wild type as long as glucose was present (Fig. 2C), the *a*H_185_R mutant divided less rapidly than the other strains in the ethanol-dependent growth phase at 36°C. Consistently, this mutant showed a significantly reduced efficiency to grow in liquid glycerol (another substrate that, like ethanol, cannot be fermented), whereas much smaller differences were observed with the seven other mutants relative to wild-type yeast (Fig. 2C).

### Influence of the *atp6* mutations on the assembly/stability of ATP synthase

**Fig. 3.**
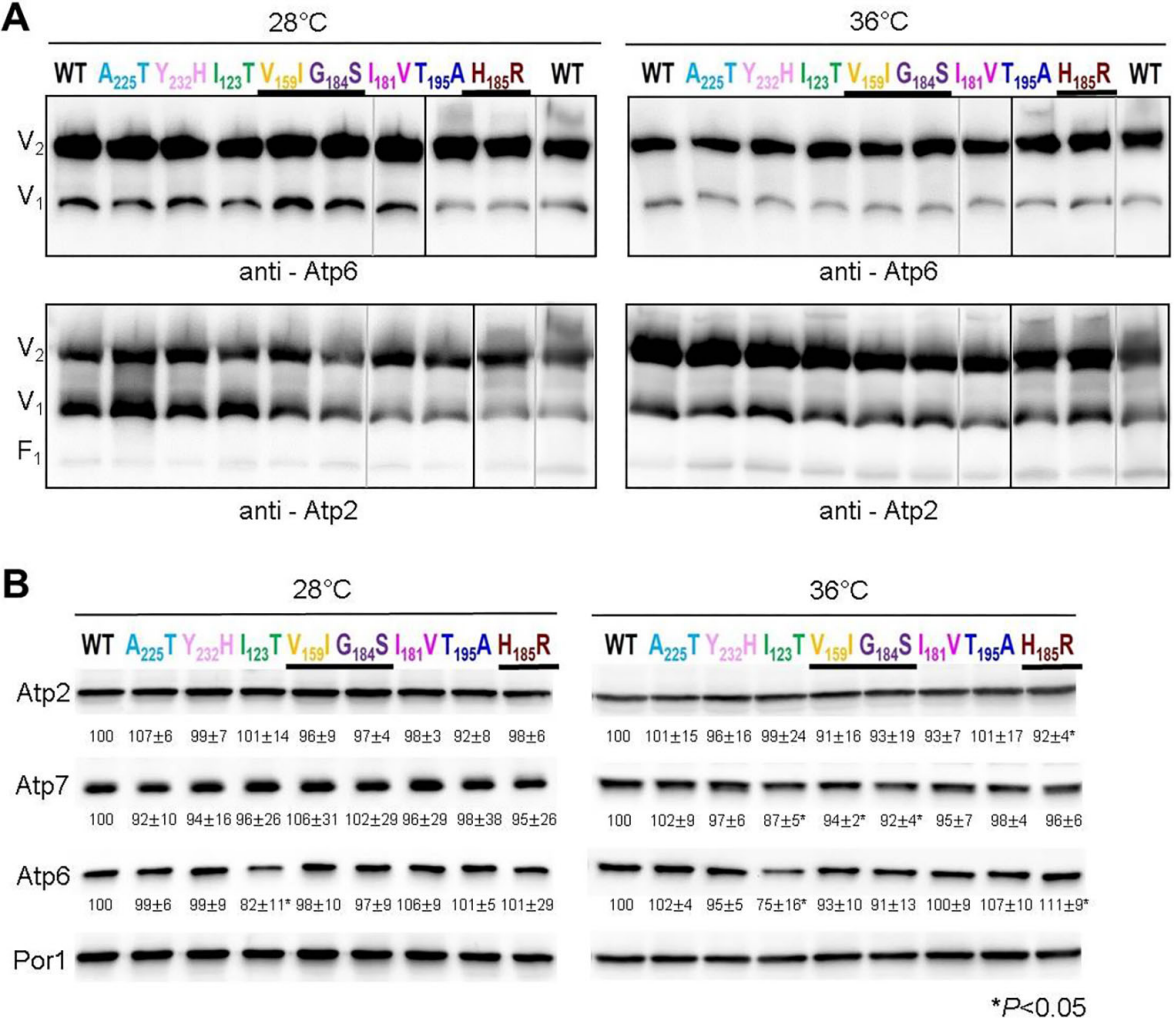
**Influence of *atp6* mutations on ATP synthase assembly/stability.** (A) Mitochondria isolated from the *atp6* mutants and wild-type yeast grown at the indicated temperature were solubilized with digitonin (1.5 g/g protein), and 200 μg of proteins were separated in BN gels containing a 3-12% polyacrylamide gradient. The proteins were transferred to a PVDF membrane and probed with antibodies against subunits *a* (Atp6) and Atp2 (β-*F*_1_) of ATP synthase. The immunological signals corresponding to dimers (V_2_) and monomers (V_1_) of ATP synthase and free *F*_1_ particles are indicated. Lanes from the same gel are demarcated by black borders, and the control lane (WT) from each gel is shown. Gray lines separate lanes that were not loaded side by side. (B) Total cellular protein extracts were separated by SDS-PAGE and then transferred to a nitrocellulose membrane and probed with antibodies against the indicated proteins. The intensity of bands was calculated using ImageJ, normalized to porin, and the differences are expressed as a percentage relative to the control strain. *P*-values and s.e.m. were calculated from three independent experiments. *P<0.05 (unpaired two-tailed Student's *t*-test).

The influence of the *atp6* mutations on ATP synthase assembly/stability was evaluated by blue native (BN)-polyacrylamide gel electrophoresis (PAGE) of mitochondrial digitonin extracts prepared from cells grown in rich galactose medium (YPGalA). In samples from the wild type, using antibodies specific to its subunits *a* and Atp2 (β-*F*_1_), ATP synthase was detected mainly as dimers and monomers, with only trace amounts of free *F*_1_ particles not associated with F_O_ (Fig. 3A). A similar pattern was observed in samples from the eight *atp6* mutants. There was no important difference in the levels of ATP synthase subunits Atp2, Atp7 (a subunit of the peripheral arm of ATP synthase) and Atp6 in total cellular protein extracts separated in denaturing gels (Fig. 3B). About a 18-25% decrease in the amount of the *a*I_123_T subunit was associated with increased amounts of mtDNA-deficient cells in cultures of these mutant cells (see Table 2). These data indicate that the mutations had little, if any, effect on the assembly/stability of subunit *a* within ATP synthase.

**Table 2.**
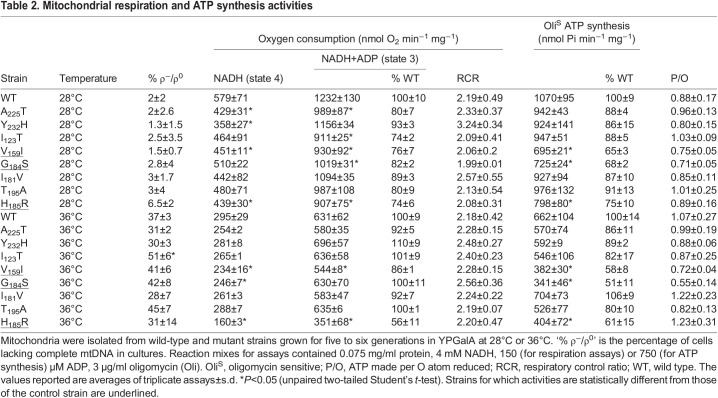
Mitochondrial respiration and ATP synthesis activities

### Respiration and ATP synthesis activities

The influence of the *atp6* mutations on oxygen consumption and ATP synthesis was investigated in intact (osmotically protected) mitochondria isolated from cells grown in galactose-rich medium at 28°C and 36°C. Oxygen consumption was measured using NADH as an electron donor. In yeast, in which there is no Complex I, electrons from NADH added to mitochondria are transferred to ubiquinone without any proton pumping by dehydrogenases (Nde1/2) located at the external surface of the inner membrane and are next transferred to oxygen, as in humans, by the proton-translocating Complexes III (*bc*_1_) and IV (*aa*_3_) (Michel, 1998; Schultz and Chan, 2001; Berry et al., 2000). With the addition of NADH alone (basal or state 4 respiration), the rate of electron transfer is mainly controlled by the passive permeability to protons of the inner membrane. With mutations that induce proton leaks through the F_O_, state 4 respiration will increase (Duvezin-Caubet et al., 2006). None of the investigated mutations stimulated state 4 respiration (Table 2), indicating the absence of such proton leaks. After a subsequent addition of ADP (state 3 respiration), the rate of electron transfer to oxygen is normally about twice stimulated to maintain across the membrane the proton gradient that is now used by the ATP synthase to produce ATP (Hinkle, 2005). Consistent with the quite good growth of the mutants on glycerol (Fig. 2A), their mitochondria responded quite well to the addition of ADP. However, a significant decrease in the rate of oxygen consumption, up to 55% (depending on the growth temperature), was observed for *a*H_185_R relative to that in wild-type yeast (Table 2). Consistently, the mitochondria from this mutant showed a similarly reduced rate of ATP synthesis. The reduced capacity of this mutant to transfer electrons to oxygen is a secondary consequence of a defect in ATP synthase and not the inverse, as was observed and discussed in previous studies (Kabala et al., 2014; Kucharczyk et al., 2019b, 2013, 2009b). Oxygen consumption at state 3 was reduced in mitochondria of *a*G_184_S and *a*V_159_I by ∼15-24%, while the rate of ATP synthesis was reduced by ∼30-51%, with statistical significance. The compromised ability of the *a*H_185_R, *a*G_184_S and *a*V_159_I cells to produce ATP in mitochondria corroborates their higher sensitivity to oligomycin when grown from non-fermentable substrates (Fig. 2B). The differences in ATP synthesis seen with the five other mutations investigated in this study were not statistically significant (Table 2), indicating that they had only minor, if any, detrimental effects on ATP synthase.

**Fig. 4.**
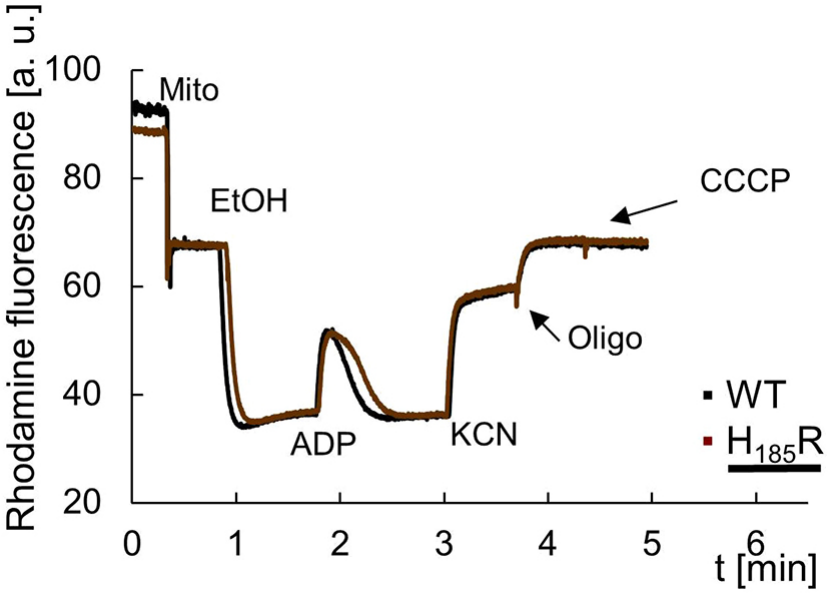
**Variations in mitochondrial inner membrane potential in *a*H_185_R mitochondria isolated from cells grown at 36°C.** The tracings show how the mitochondria responded to externally added ADP. The additions were 75 μM ADP, 0.5 μg/ml Rhodamine 123, 75 μg/ml mitochondrial proteins (Mito), 10 μl ethanol (EtOH), 2 mM potassium cyanide (KCN), 4 μg/ml oligomycin (Oligo) and 4 μM carbonyl cyanide-m-chlorophenyl hydrazone (CCCP). The shown tracings are representative of three biological repetitions. a.u., arbitrary units.

We further evaluated the influence of substitutions in subunit *a* on oxidative phosphorylation by monitoring changes in the mitochondrial transmembrane potential (ΔΨ). In a first series of experiments (Fig. 4; Fig. S1A), we tested the capacity of ADP to induce ΔΨ in ethanol-energized mitochondria due to proton re-entry through the *F*_1_F_O_-ATP synthase, followed by a total collapse of the membrane potential with further additions of potassium cyanide (KCN) and carbonyl cyanide m-chlorophenylhydrazone (CCCP). Mitochondria from all strains responded similarly to the control mitochondria except those from *a*H_185_R cells grown at elevated temperature, which needed more time to re-establish the potential after addition of ADP. We next investigated the ability of ATP synthase to rebuild the ΔΨ when working in the reverse mode (Fig. S1B). The mitochondria were energized by ethanol, the resulting ΔΨ was collapsed with KCN, and ATP was then rapidly added. In these conditions, the natural *F*_1_ inhibitor protein IF1 is released from *F*_1_ and does not re-bind (Venard et al., 2003). In mitochondria of all strains, ATP addition induced a large and stable ΔΨ that was fully reversed by oligomycin.

## DISCUSSION

**Fig. 5.**
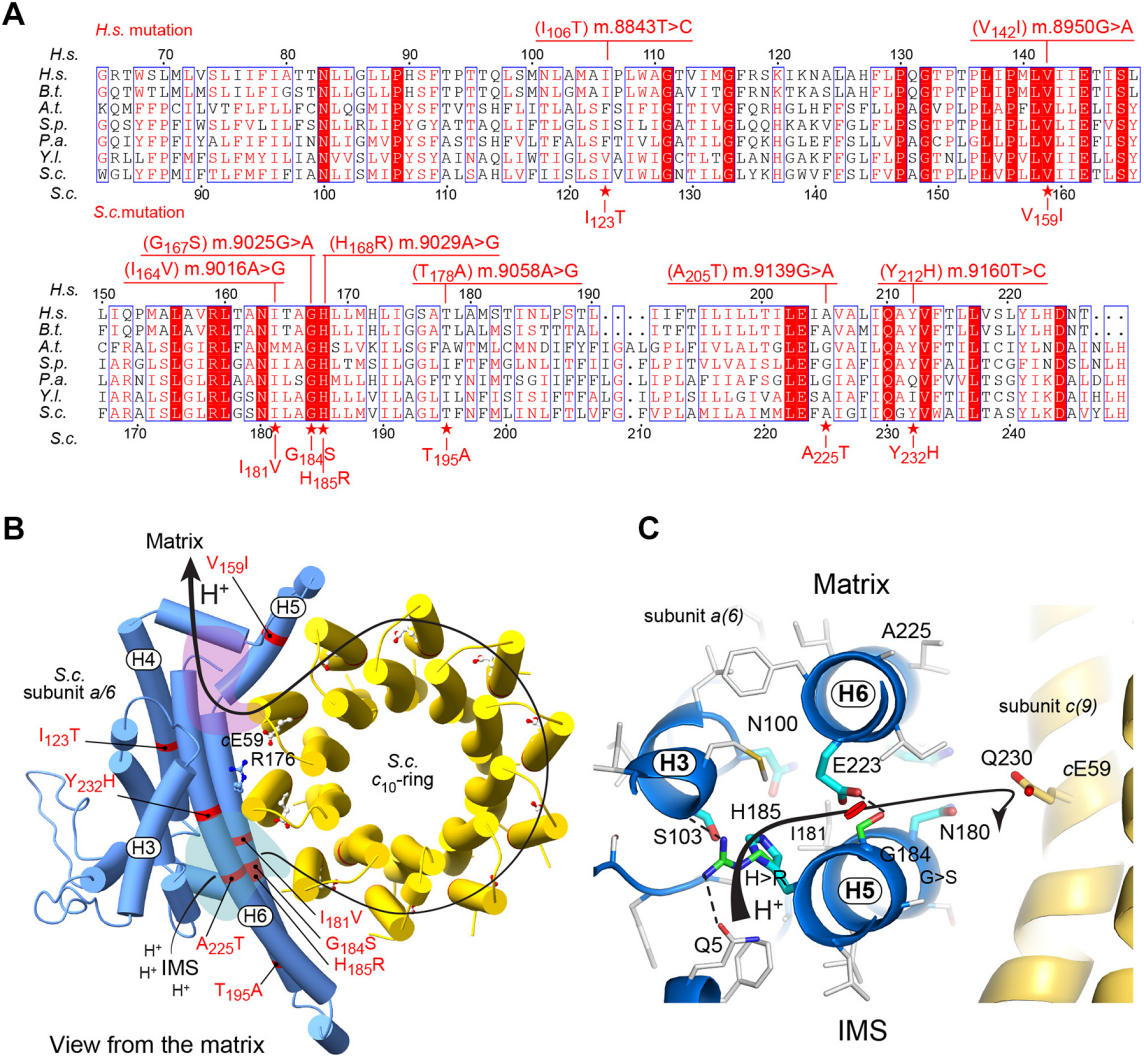
**Evolutionary conservation and topology of the subunit *a* residues targeted by the mutations investigated in this study.** (A) Alignments of the regions of human (*H.s.*) subunit *a* in which the mutations investigated in this study are located, with their counterparts in *Bos taurus* (*B.t.*), *Arabidopsis thaliana* (*A.t.*), *Schizosaccharomyces pombe* (*S.p.*), *Podospora anserina* (*P.a.*), *Yarrowia lipolytica* (*Y.l.*) and *Saccharomyces cerevisiae* (*S.c.*). The positions of the residues in *H.s.* and *S.c.* are indicated above and below the alignments, respectively. The positions of the nucleotide changes in human mtDNA and the amino acid changes they induce, as well as the corresponding amino acid changes in the yeast protein, are indicated. (B) Overall top view from the mitochondrial matrix of the *S. cerevisiae a*/*c*_10_-ring structure with the pathway along which protons are moved from the intermembrane space (IMS) to the mitochondrial matrix. The *n*-side and *p*-side hydrophilic pockets involved in this transfer are colored in purple and pale green, respectively. The E_59_ in subunit *c* and R_176_ in subunit *a* that are essential for F_O_-mediated proton transfer are shown as yellow and blue sticks, respectively. The positions of the eight mutations investigated in this study are indicated. (C) Detailed view of the *p*-side pocket and the subunit *c* towards which a proton is transferred from the IMS. Important hydrophilic side chains are drawn as sticks colored in cyan. Those two amino acid changes (G_184_S and H_185_R) that compromise proton translocation are drawn as sticks with their carbon in green color.

It is in the *MT-ATP6* gene encoding the subunit *a* of ATP synthase that the first pathogenic mutation of mtDNA, m.8993T>G, was identified >30 years ago (Holt et al., 1990). Since then, dozens of mutations of this gene were detected in patients with various neuromuscular diseases (Dautant et al., 2018). With the recent advent of complete structures of ATP synthase from various mitochondrial origins (Guo et al., 2017; Hahn et al., 2016; Sobti et al., 2020; Spikes et al., 2020), we better understand the mechanism of proton transport through the F_O_ domain of the enzyme, and we can analyze how amino acid substitutions disrupt the functioning of the enzyme. The subunit *a* and a ring of identical subunits *c* (eight in humans, ten in yeast) are responsible for the transport of protons across the membrane domain (F_O_) of ATP synthase (Fig. 5). Two hydrophilic pockets on the external (*p*) and internal (*n*) sides of the inner membrane allowing access to the contact zone between the *c*-ring and subunit *a* enable the transfer of protons from the intermembrane space of mitochondria to an acidic residue in subunit *c* (*c*E_59_ in yeast) and their release in the mitochondrial matrix after an almost complete rotation of the *c*-ring (Guo et al., 2017). The *p*- and *n*-side pockets are separated by a plug of hydrophobic residues of subunit *a* near the middle of the membrane, and close to it, in front of *c*E_59_, is a positively charged arginine residue (*a*R_176_ in yeast) that is essential for moving protons through the F_O_ (Guo et al., 2017). Of the eight mutations herein investigated, two *a*H_185_R and *a*G_184_S that significantly impacted ATP synthase function are within the *p*-pocket. It can be inferred from mutagenesis studies in *Escherichia coli* (Cain and Simoni, 1988; Lightowlers et al., 1988), and atomic structures of bacterial and mitochondrial ATP synthases (Guo et al., 2017; Sobti et al., 2020; Srivastava et al., 2018), that the *a*H_185_ residue of yeast subunit *a* is most likely involved in F_O_-mediated proton transfer in concert with a nearby glutamate residue (*a*E_223_ in yeast), owing to the ability of these two residues to exchange protons (Fig. 5C). Although it can also exchange protons owing to its guanidinium group, the side-chain of *a*R_185_ is, according to our structural modeling analyses, more distantly located from *a*E_223_ than the imidazole group of *a*H_185_. Additionally, *a*R_185_ has the ability to make hydrogen bonds with a glutamine residue (*a*Q_5_) located near the N-terminal end of subunit *a* (Fig. 5C). Therefore, the replacement of histidine in position 185 with arginine will impair the function of *a*E_223_ in the transport of protons. The lack of a side chain at position 184 (owing to its occupation by a glycine residue) is presumably important to avoid steric hindrance between *a*H_185_ and *a*E_223_ (Fig. 5C). In our structural analyses, the hydroxyl group of the serine side-chain in position 184 can make a hydrogen bond with *a*E_223_ and thereby compromise its proton-conduction function within the *p*-pocket. Although the six other *MT-ATP6* gene variants herein investigated lead to replacement of well-conserved subunit *a* residues (*a*I_123_T, *a*V_159_I, *a*I_181_V, *a*T_195_A, *a*A_225_T and *a*Y_232_H, in the yeast protein), these are in positions remote from the regions at the *a*/*c* interface that are critical for F_O_-mediated proton transport (Fig. 5B,C). These changes do not induce steric or charge hindrance in their proximal environment.

If the structural analysis considerably helps making predictions on how discrete structural changes in subunit *a* may influence the stability/activity of ATP synthase, and hence human health, a genetically approachable model system is required to test such predictions. The yeast *S. cerevisiae* is a particularly convenient system for investigations of substitutions in mitochondrially encoded subunits that are highly conserved, like subunit *a* of ATP synthase (Fig. 5A). Its mitochondrial genome can be manipulated, and heteroplasmy is highly unstable in this organism. Taking advantage of these unique attributes, we were able to define how ten *ATP6* mutations known to be pathogenic affect the ATP synthase (Ding et al., 2020; Kabala et al., 2014; Kucharczyk et al., 2019a,b, 2010, 2013, 2009a,b,c; Skoczen et al., 2018; Su et al., 2020, 2021). In this study, we investigated, in yeast, eight additional missense *MT-ATP6* mutations recently identified in patients, for which there is little understanding of how these mutations cause disease.

Four of these mutations (m.8950G>A, m.9016A>G, m.9029A>G and m.9139G>A) were found in a very small number of patients (one or two individuals only) presenting with clinical features typical of Leber hereditary optic neuropathy (LHON), a disease characterized by optic nerve degeneration and loss of vision (Finsterer et al., 2018). Thus far, LHON-causing mutations were mostly located in the mitochondrial MT-ND genes encoding subunits of Complex I. Although they affect highly conserved residues of subunit *a*, equivalents of m.9016A>G and m.9139G>A had no obvious detrimental effects on the activity and assembly/stability of the yeast ATP synthase, which casts in doubt their potential, at least alone, to impair vision. Consistently, the amino acid replacements they induce are not in regions of subunit *a* known to be critical for moving protons across the membrane domain of ATP synthase (Fig. 5). The health problems of the individuals with m.9016A>G and m.9139G>A are thus likely to be caused by the primary mtDNA variants leading to LHON, also found in those two patients: m.14484T>C mutation in *MT-ND6* and m.11778G>A mutation in *MT-ND4* genes, respectively (La Morgia et al., 2008; Povalko et al., 2005). We cannot rule out, however, that they might have some detrimental effects if combined with other mutations in nuclear DNA or mtDNA that affect the process of oxidative phosphorylation, as was documented for a number of other mutations of the human mtDNA (Ban et al., 2008; McManus et al., 2019). In contrast, the m.8950G>A variant was found in two unrelated patients: alone in a female with LHON plus dystonia (Abu-Amero and Bosley, 2005) and in combination with the m.13513G>A leading to p.D_393_N substitution in the ND5 subunit of the respiratory chain complex I in an individual with Leigh-like syndrome (Brautbar et al., 2008). The family history and biochemical data from patients cells are missing; thus, it is unclear why this mutation was classified as benign in the ClinVar database (Table 1). Substitution of *a*V159 into I in yeast Atp6 protein inhibited the growth of yeast cells on respiratory medium in the presence of oligomycin and decreased the ATP production rate by ∼35%. Our data indicate that this mutation alone may have led to LHON in the patient described in Abu-Amero and Bosley (2005) and could have aggravated the severity of Leigh disease in the patient described in Brautbar et al. (2008), but more patient data are needed to classify this variant as pathogenic.

Regarding the fourth *MT-ATP6* variant (m.9029A>G), detected in an atypical LHON patient (with progressive loss of vision over 4 years) and in a second patient diagnosed with mitochondrial disorder, there is some evidence that it might have detrimental effects on mitochondrial function and was therefore suspected to be pathogenic (Lopez-Gallardo et al., 2014). The present study strongly supports this proposal. Indeed, the growth of yeast cells with an equivalent of this mutation (*a*H_185_R) on respiratory carbon sources was less efficient than that in wild-type cells and was highly sensitive to chemical inhibition of F_O_ with oligomycin, indicating a substantial loss of ATP synthase function; this was confirmed by direct ATP synthesis activity measurements in isolated mitochondria. Based on previous studies in *E. coli* (Cain and Simoni, 1988; Lightowlers et al., 1988) and the results reported here, we suggest that *a*H_185_R compromises the transport of protons from the mitochondrial intermembrane space to the *c*-ring motor of ATP synthase.

The four remaining variants here investigated were identified in patients presenting with schizophrenia (m.8843T>C, m.9160T>C), maternally inherited Leigh syndrome (MILS) (m.9025G>A) or left ventricular hypertrabeculation syndrome (LVHT) (m.9058A>G) (Table 1). Of these, only m.9025G>A had clear detrimental effects in a yeast model of this mutation (*a*G_184_S), as evidenced by a higher *in vivo* sensitivity to oligomycin (Fig. 2A,B) and a substantial drop in the rate of mitochondrial ATP synthesis relative to that in wild-type yeast (Table 2). This variant was found in three independent patients with MILS or motor neuropathy but was also found in 13 healthy individuals, and in homoplasmy in the asymptomatic mother and sister of one of the above patients (Lopez-Gallardo et al., 2014); therefore, it was classified as benign in the ClinVar database. However, the pathogenicity score of this variant is very high (Pereira et al., 2011), and another substitution of glycine 167 – into lysine – owing to m.9026G>A was found in two members of one family with intellectual disability, dysautonomia, headaches, myalgia and fatigue (Ganetzky et al., 2019). The p.G_167_K substitution was proposed to be pathogenic based on fibroblast respirometry and family genetics. These findings, and the data we obtained in the yeast model, provide arguments for the pathogenicity of the m.9025G>A variant as well. As we described above, this mutation possibly prevents a glutamate residue (*a*E_223_) from functioning properly in the transfer of protons within the *p*-side proton channel of F_O_.

Although equivalents of m.8950G>A and m.9025G>A had significant detrimental effects in yeast, these variants were seen at homoplasmy in >40 healthy individuals in the gnomAD population database. This raises the possibility that, in those individuals showing a clinical phenotype, these variants act in synergy with some additional genetic defect in nuclear DNA, as was previously observed for other mtDNA mutations (Dunham-Snary et al., 2018; Pickett et al., 2018). Also, it is well known that some mutations in mtDNA start to compromise human health in older age. This was, for instance, observed in a patient who, at the age of 68, developed a common mitochondrial disorder due to a mutation in the *MT-ATP6* gene (J.P.d.R. and V. Procaccio, University Hospital of Angers, Angers, France, personal communication). It would be interesting to follow up those healthy individuals carrying m.8950G>A or m.9025G>A when they become aged.

The good preservation of ATP synthase function in yeast strains with equivalents of m.8843T>C (*a*I_123_T), m.9160T>C (*a*Y_232_H) and m.9058A>G (*a*T_195_A) suggests that these mutations do not have, at least alone, the potential to compromise human health.

Thanks to their unique features, such as the ability to fermentative growth, the possibility of mtDNA mutagenesis and its homoplasmy, yeast *S cerevisiae* is a very good model for understanding the pathogenesis of mutations in genes encoded in mtDNA. However, the usefulness of yeast is limited to highly evolutionarily conserved proteins like Atp6, but less so for Atp8, which shows great diversity at the sequence level. Although the analyses of many of the variants we have examined so far in this model correlate with the severity of the disease in patients, information on family history, and studies’ on patients cells or tissues, are necessary to unequivocally classify mitochondrial variants as pathogenic.

## MATERIALS AND METHODS

### Media for growing yeast

The media used for growing yeast were as follows: YPGA (1% Bacto yeast extract, 1% Bacto Peptone, 2% or 10% glucose, 40 mg/l adenine), YPGalA (1% Bacto yeast extract, 1% Bacto Peptone, 2% galactose, 40 mg/l adenine), YPGlyA (1% Bacto yeast extract, 1% Bacto Peptone, 2% glycerol, 40 mg/l adenine), BIOL-Leu [1.7 g/l YNB w/o (yeast nitrogen base without amino acids), 5 g/l ammonium sulfate, 0.8 g/l CSM-Leu drop out mix, 5% glucose, 182.5 g/l sorbitol and 40 mg/l adenine]. Media were solidified by the addition of 2-5% (w/v) Bacto agar. Growth curves were established with a Bioscreen CTM system. For the drop test, cells were grown in liquid YPGA medium for one night. The density of cultures was measured, and they were serially diluted in water to have, in the fifth dilution, ten colonies in a 5 µl drop. Then, the drops were added to the plates. Growth tests were performed at least two times. For halo tests, exponentially grown cells, at an optical density at a wavelength of 600 nm (OD_600_) of 0.05, were homogeneously spread with sterile glass beads on a Petri dish containing solid YPGlyA medium. Sterile filters were deposited on the plate and spotted with 2 µl of 10 mM oligomycin dissolved in dimethyl sulfoxide (DMSO). The diameter of the growth inhibition halo, a value permitting the comparison of strains for their sensitivity to oligomycin, was measured with the ImageJ program.

### Construction of the yeast *atp6* mutants

Q5^®^ and QuikChange II site-directed mutagenesis kits (from NEBiolabs and Agilent, respectively) were used for mutagenizing the yeast *ATP6* gene (Tribouillard-Tanvier et al., 2022). To this end, three previously described plasmids were used: pSDC8 (Zeng et al., 2007), pSDC9 (Kucharczyk et al., 2009b) and pSDC14 (Rak et al., 2007a). Plasmid pSDC8 contains, within a *Bam*HI-*Eco*RI fragment, the first 698 bp of the yeast *ATP6* gene, while the remaining 82 bp are within an *Eco*RI-*Bam*HI fragment in pSDC9, and pSDC14 results from cloning of the whole *ATP6* gene at the *Bam*HI site of plasmid pJM2 (Fig. 1). Equivalents of the m.8843T>C, m.8950G>A, m.9016A>G, m.9025G>A, m.9029A>G and m.9058A>G mutations (*atp6^mut^*) were introduced in pSDC8, and equivalents of m.9139G>A and m.9160T>C were created in pSDC9 (see Table S1 for the sequences of the mutagenic primers and Table 1 for the corresponding codon and amino acid changes). The mutated *ATP6* fragments in pSDC8 and pSDC9 were cut off (with *Bam*HI+*Eco*RI and *Eco*RI+*Sap*I, respectively) and exchanged with the corresponding (non-mutated) fragments in pSDC14, yielding the final plasmids with the *atp6^mut^* mutations in the entire *ATP6* gene (pATP6^mut^). These plasmids, together with a plasmid (Yep351) carrying the nuclear *LEU2* gene, were introduced into the ρ^0^ strain DFS160 by microprojectile bombardment using a biolistic PDS-1000/He particle delivery system (Bio-Rad) as described (Bonnefoy and Fox, 2001). The Leu^+^ transformants (a few hundred per plate) were cross-replicated during one night on a rich glucose medium (YPGA) with cells from strain MR10, in which the coding sequence of *ATP6* is replaced by the *ARG8^m^* genetic marker (Rak et al., 2007b). In these crosses, the Leu^+^ transformants that contain the pATP6^mut^ plasmid (ρ^−^ *atp6^mut^*) in their mitochondria are able, by mtDNA recombination, to replace *ARG8^m^* by the mutated *ATP6* gene, yielding strains in which the *atp6* mutation is integrated in a complete mitochondrial genome (ρ^+^ *atp6^mut^*). If the *atp6^mut^* does not fully inactivate the ATP synthase, the ρ^+^ *atp6^mut^* clones can be selected by their ability to grow on glycerol medium (YPGlyA). Respiring clones were obtained with the eight pATP6^mut^ plasmids, and the presence in these clones of the *atp6^mut^* mutations was confirmed by DNA sequencing with primers oATP6-1 and oATP6-10 (Table S1).

### Biochemical analyses of mitochondria

Mitochondria were prepared from yeast cells grown in rich galactose (YPGalA) to an OD_600_ of 4 by the enzymatic method described in the reference (Guerin et al., 1979). For respiration and ATP synthesis assays, they were diluted to 0.075 µg/ml in respiration buffer (10 mM Tris-maleate pH 6.8, 0.65 M mannitol, 0.36 mM EGTA and 5 mM Tris-phosphate). Oxygen consumption rates were measured using a Clarke electrode in the presence of 4 mM NADH (state 4 respiration), 150 µM ADP (state 3 respiration) or 4 µM CCCP (uncoupled respiration), as previously described (Rigoulet and Guerin, 1979). A higher concentration of ADP (750 µM) was used to measure the rate of ATP synthesis in the absence and presence (3 μg/ml) of oligomycin. Aliquots were taken every 15 s, and the production of ATP, after stopping the reaction with 3.5% (w/v) perchloric acid and 12.5 mM EDTA, was quantified using Kinase-Glo Max Luminescence Kinase Assay (Promega) and a Beckman Coulter's Paradigm Plate Reader. Variations in ΔΨ were evaluated in the respiration buffer containing 0.150 mg/ml mitochondria and Rhodamine 123 (0.5 μg/ml), with an excitation wavelength of 485 nm and an emission wavelength of 533 nm under constant stirring using a Cary Eclipse Fluorescence Spectrophotometer (Agilent Technologies) (Emaus et al., 1986). BN-PAGE was performed as described (Schagger and von Jagow, 1991) from mitochondrial samples containing 200 µg of proteins suspended in 100 µl extraction buffer [30 mM HEPES pH 6.8, 150 mM potassium acetate, 12% glycerol, 2 mM 6-aminocaproic acid, 1 mM EGTA, 1.5% digitonin (Sigma-Aldrich)]. To avoid protein degradation, one protease inhibitor cocktail tablet (Roche) per 10 ml and 1 mM PMSF were added to the samples. After 26 min incubation on ice, the extracts were cleared by centrifugation (21,950 ***g***, 4°C, 30 min), supplemented with 4.5 µl loading dye (5% Serva Blue G-250, 750 mM 6-aminocaproic acid) and run on NativePAGE^TM^ 3-12% Bis-Tris Gels (Thermo Fisher Scientific). After transfer onto a PVDF membrane, ATP synthase complexes were detected using polyclonal antibodies against yeast subunit *β* (Atp2p) or *a* (Atp6p) at a 1:10,000 dilution. For sodium dodecyl-sulfate (SDS)-PAGE analysis, 10 OD_600_ of overnight grown cells in YPGalA medium was centrifuged and suspended in 500 µl of 0.2 M NaOH. After 10 min incubation on ice, the samples were mixed with 50 µl of 50% trichloroacetic acid, incubated for 10 min on ice, and centrifuged at 21,950 ***g*** for 10 min at 4°C. The protein pellet was washed with 1 ml of 1 M Tris-base and suspended in 50 µl of 5% SDS, and the concentration of proteins was measured by the method of Lowry (Lowry et al., 1951). For those samples prepared from cells grown at 28°C or 36°C, 40 µg and 75 µg of proteins, respectively, were run on 12% SDS-PAGE gels (Laemmli, 1970). The proteins were then transferred onto a nitrocellulose membrane using an iBlot2 Gel Transfer Device from Thermo Fisher Scientific and analyzed by western blotting using antibodies against Atp2, Atp6, Atp7 and Por1 [these were kindly provided by Marie-France Giraud (Institut de Biochimie et Génétique Cellulaires, Centre National de la Recherche Scientifique, France; 1:10,000) and Teresa Żołądek (Institute of Biochemistry and Biophysics, Polish Academy of Sciences, Warsaw, Poland; 1:10,000)].

### Amino acid alignments and topology of subunit *a* mutations

Multiple sequences of ATP synthase *a*-subunits of various origins were aligned and drawn using Clustal Omega (Sievers et al., 2011) and Espript 3.0 (Robert and Gouet, 2014), respectively. Molecular views of subunit *a* and *c*10-ring were obtained from the dimeric Fo domain of *S. cerevisiae* ATP synthase [PDB ID: 6b2z (Guo et al., 2017)]. The structure figure was drawn using PyMOL molecular graphic system.

### Statistical analysis

At least three biological and three technical replicates were performed for all experiments. Unpaired two-tailed Student's *t*-test was used for all datasets. Significance and confidence level was set at 0.05.

### Statement of ethics

The permission number for work with genetically modified microorganisms (GMM I) for R.K. is 01.2-28/201.

## Supplementary Material

10.1242/dmm.049783_sup1 Supplementary information
