# Supplementary material for: Probing the pathogenicity of patient-derived variants of MT-ATP6 in yeast
Source: Dis Model Mech. 2023 Apr 21;16(4):dmm049783. doi: 10.1242/dmm.049783 (PMC10151828; doi:10.1242/dmm.049783)
Supplement: Supplementary information [file dmm-16-049783-s1.pdf]

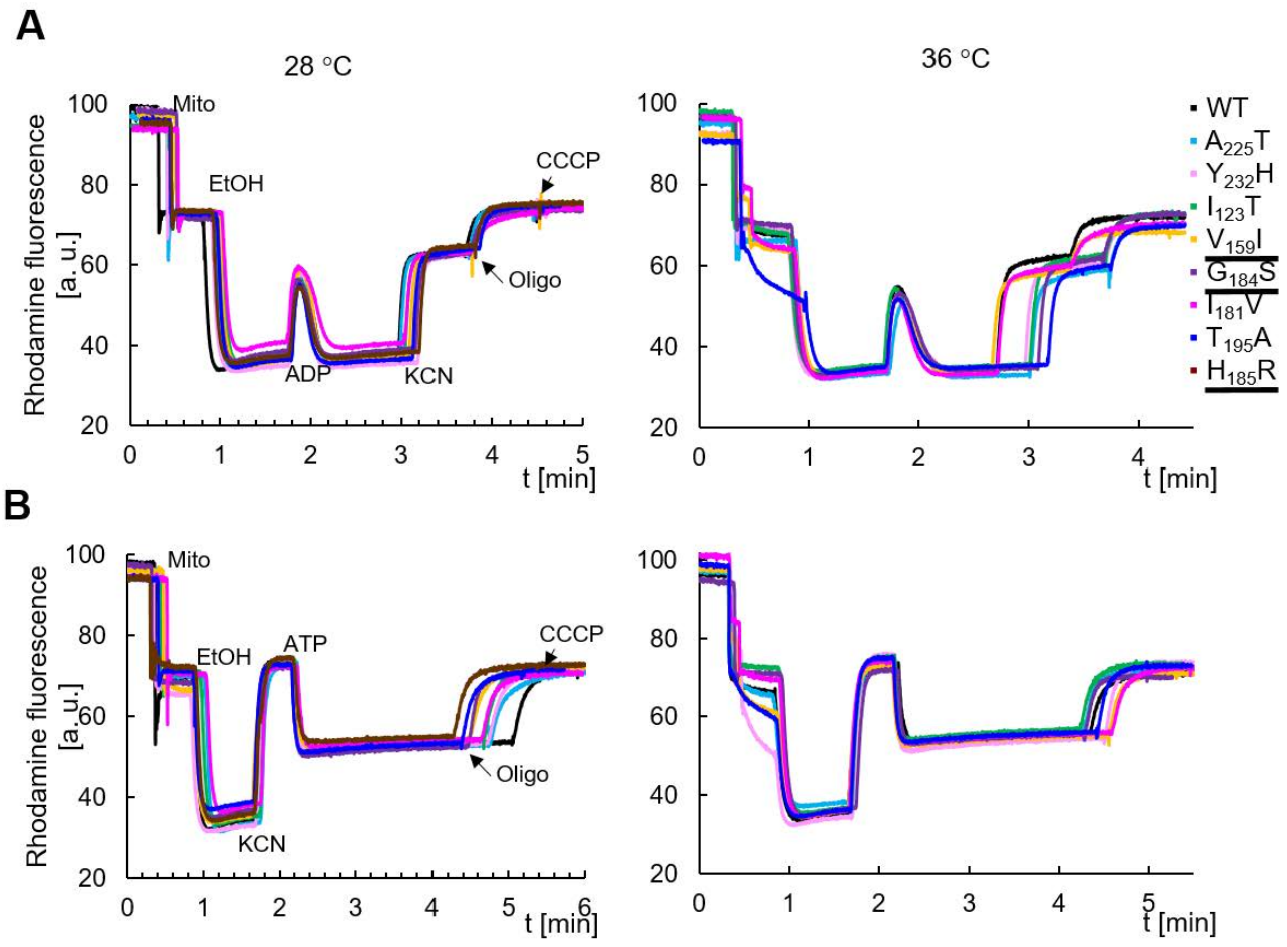

Fig. S1. Variations in mitochondrial membrane potential. The tracings in panel A show how the mitochondria responded to externally added ADP, those in panel B reflect ATP-driven proton pumping by ATP synthase. The additions were 0.5 µg/ml Rhodamine 123, 75 µg/m mitochondrial proteins (Mito), 10 µL ethanol (EtOH), 75 µM ADP, 0.2 mM ATP, 2 mM potassium cyanide (KCN), 4 µg/ml oligomycin (oligo), and 4 µM carbonyl cyanide-m-chlorophenyl hydrazone (CCCP). The shown tracings are representative of three biological repetitions.

Disease Models & Mechanisms • Supplementary information

Table S1. List of oligonucleotides used in the study (in bold are the mutagenic nucleotides)

Amino acid change in Atp6p	Codon change in *ATP6* gene	Primers		pSDC14 based plasmid	pATP6^{mut} plasmid
I$_{123}$T	ATT>ACT	For	GTATGATTCCATACTCATTTGCATTATCAGCT	pM6.1	pEB12
		Rev	CTAAAATAGTATTACCTAATCAAATAACA**G**TACTTAAAGAGATAATAAATACTAAATG		
V$_{159}$I	GTT>ATT	For	GTATTCTTCTCATTATTCGTACCTGCTGGTA	pN6.1	pEB13
		Rev	GCGAAATAAGATAAAGTTTCAATAATAA**T**TAATAAAGGTACTAATGGTAATGGTG		
I$_{181}$V	ATC>GTC	For	GAAACTTTATCTTATTTCGCTAGAGCTATTT	pO73.1	pEB20
		Rev	ATAACCATTAATAAATGACCAGCTAAGA**C**ATTAGAACCTAATCTTAAACCTAATG		
G$_{184}$S	GGT>AGT	For	CTTATTTCGCTAGAGCTATTTCATTAGGTT	pP5.1	pEB14
		Rev	CCAGCTAAAATAACCATTAATAAATGAC**T**AGCTAAGATATTAGAACCTAATCTTA		
H$_{185}$R	CAT>AGA	For	CTTATTTCGCTAGAGCTATTTCATTAGGTTTAA	pQ53.9	pEB21
		Rev	GTAAACCAGCTAAAATAACCATTAATAA**TCT**ACCAGCTAAGATATTAGAACCTAATC		
T$_{195}$A	CTA>GCA	For	TATTAATGGTTATTTTAGCTGGTTTA**GC**ATTTAATTTTATGTTAATTAATTTATTTAC	pT4.1	pEB17
		Rev	CATAGCTAAAGGTACAAAACCGAATACTAAA		
A$_{225}$T	GCT>ACT	For	CACGACGTTGTAAAACGACGGCCAGTGAATTC**A**CTATTGGTATCATTCAGGGATATGTCTG	-	pEB10
		Rev	CAGACATATCCCTGAATGATACCAATAG**T**GAATTCACTGGCCGTCGTTTTACAACGTCGTG		
Y$_{232}$H	TAT>CAT	For	GAATTCGCTATTGGTATCATTCAGGGA**C**ATGTCTGGGCTATTTTAACAGCATCATA	-	pEB11
		Rev	TATGATGCTGTTAAAATAGCCCAGACAT**G**TCCCTGAATGATACCAATAGCGAATTC		
oATP6-1	-	-	TAATATACGGGGGTGGGTCCCTCAC	-	-
oATP6-10	-	-	GGGCCGAACTCCGAAGGAGTAAG	-	-

Disease Models & Mechanisms • Supplementary information

Table S2. Genotypes and sources of yeast strains

Strain	Nuclear genotype	mtDNA	Source
DFS160	*MATα leu2Δ ura3-52 ade2-101 arg8::URA3 kar1-1*	ρ^0	Steele et al., 1996
NB40-3C	*MAT**a** lys2 leu2-3,112 ura3-52 his3ΔHinDIII arg8::HIS3*	ρ^+ *cox2-62*	Steele et al., 1996
MR6	*MAT**a** ade2-1 his3-11,15 trp1-1 leu2-3,112 ura3-1 arg8::HIS3*	ρ^+	Rak et al., 2007b
MR10	*MAT**a** ade2-1 his3-11,15 trp1-1 leu2-3,112 ura3-1 arg8::HIS3*	ρ^+*atp6::ARG8* ^{m}	Rak et al., 2007b
SDC30	*MATα leu2Δ ura3-52 ade2-101 arg8::URA3 kar1-1*	ρ^- *ATP6 COX2*	Rak et al., 2007a
EBY1a	*MATα leu2Δ ura3-52 ade2-101 arg8::URA3 kar1-1*	ρ^- *atp6*-A225T	This study
EBY2a	*MATα leu2Δ ura3-52 ade2-101 arg8::URA3 kar1-1*	ρ^- *atp6*-Y232H	This study
EBY3a	*MATα leu2Δ ura3-52 ade2-101 arg8::URA3 kar1-1*	ρ^- *atp6*-I123T	This study
EBY4a	*MATα leu2Δ ura3-52 ade2-101 arg8::URA3 kar1-1*	ρ^- *atp6*-V1569I	This study
EBY5a	*MATα leu2Δ ura3-52 ade2-101 arg8::URA3 kar1-1*	ρ^- *atp6*-G184S	This study
EBY8a	*MATα leu2Δ ura3-52 ade2-101 arg8::URA3 kar1-1*	ρ^- *atp6*-I181V	This study
EBY11a	*MATα leu2Δ ura3-52 ade2-101 arg8::URA3 kar1-1*	ρ^- *atp6*-T195A	This study
EBY12a	*MATα leu2Δ ura3-52 ade2-101 arg8::URA3 kar1-1*	ρ^- *atp6*-H185R	This study
EBY1	*MAT**a** ade2-1 his3-11,15 trp1-1 leu2-3,112 ura3-1 arg8::HIS3*	ρ^+ *atp6*-A225T	This study

Disease Models & Mechanisms • Supplementary information

Strain	Nuclear genotype	mtDNA	Source
EBY2	*MAT**a** ade2-1 his3-11,15 trp1-1 leu2-3,112 ura3-1 arg8::HIS3*	ρ^+ *atp6*-Y232H	This study
EBY3	*MAT**a** ade2-1 his3-11,15 trp1-1 leu2-3,112 ura3-1 arg8::HIS3*	ρ^+ *atp6*-I123T	This study
EBY4	*MAT**a** ade2-1 his3-11,15 trp1-1 leu2-3,112 ura3-1 arg8::HIS3*	ρ^+ *atp6*-V159I	This study
EBY5	*MAT**a** ade2-1 his3-11,15 trp1-1 leu2-3,112 ura3-1 arg8::HIS3*	ρ^+*atp6*-G184S	This study
EBY8	*MAT**a** ade2-1 his3-11,15 trp1-1 leu2-3,112 ura3-1 arg8::HIS3*	ρ^+ *atp6*-I181V	This study
EBY11	*MAT**a** ade2-1 his3-11,15 trp1-1 leu2-3,112 ura3-1 arg8::HIS3*	ρ^+ *atp6*-T195A	This study
EBY12	*MAT**a** ade2-1 his3-11,15 trp1-1 leu2-3,112 ura3-1 arg8::HIS3*	ρ^+*atp6*-H185R	This study

Disease Models & Mechanisms • Supplementary information
